# Chain-mediated pathways of adults socioeconomic status affecting physical fitness in Macao: the role of motor skills and moderate to vigorous physical activity

**DOI:** 10.3389/fpubh.2025.1652474

**Published:** 2025-09-01

**Authors:** Mingzhe Li, Yibo Gao, Yanfeng Zhang, Jin He, Xiaoxiao Chen, Hejie Zhang, Xiang Pan, Lupei Jiang, Haixia Hu

**Affiliations:** ^1^China Institute of Sport Science, Beijing, China; ^2^Graduate School of Health and Sports Science, Juntendo University, Inzai, Japan; ^3^College of Physical Education and Sports Rehabilitation, Jinzhou Medical University, Jinzhou, China; ^4^Weifang Aiudo Teenagers Sport & Health Institute, Weifang, China

**Keywords:** socioeconomic status, motor skills, moderate to physical activity, physical fitness, chain mediation effects

## Abstract

To investigate the relationship between adults’ socioeconomic status and physical fitness, and the chain mediation effects of motor skills and moderate to vigorous physical activity on it. Based on the 2020 Macao Special Administrative Region Citizen’s Physical Fitness and Health Surveillance data, this study was conducted with 3,695 adults aged 20–59 years, and the data were analyzed using SPSS 29.0 software. The findings showed that (1) family socioeconomic status was positively correlated with physical fitness (*p* < 0.01); (2) motor skills and moderate-to vigorous physical activity mediated the above relationship, and the mediation consisted of three pathways: the separate mediation of motor skills, the separate mediation of moderate- -to vigorous physical activity, and the chain mediation of motor skills- moderate- -to vigorous physical activity. The findings reveal that family socioeconomic status can influence adult physical fitness through three pathways: motor skills, moderate to vigorous physical activity, and motor skills-moderate-to-vigorous physical activity chain mediation, which is of great theoretical significance for the improvement of adults’ physical fitness.

## 1 Introduction

Physical Fitness (PF) is a core indicator of individual health and public health, which is not only closely associated with the risk of cardiovascular disease (1, 2), type II diabetes (3, 4) and other chronic non-communicable diseases, but also the key to slowing down aging and improving cognitive function and quality of life (5). However, the global trend of declining adult PF has become a serious public health challenge (6, 7), which also exists in China (8), creating a huge medical and economic burden for the government.

Family, as the main living scenario for adults, has the ability to influence PF in adulthood (9). Socioeconomic status (SES), which is often recognized as a composite measure of family, has been widely shown to be an important determinant of health outcomes (10, 11). Therefore, this study proposed the first hypothesis that SES has a positive predictive effect on PF.

Meanwhile, existing evidence suggests that motor skills (MS) (12, 13) and Moderate to Vigorous Physical Activity (MVPA) (14, 15) are closely associated with PF. Individuals with higher MS tend to exhibit greater self-efficacy and intrinsic motivation for physical activity, which in turn facilitates more active and sustained participation in MVPA (16). In addition, SES has been shown to be a significant influence on MS (10, 17), and MVPA (18, 19). Therefore, the second and third hypotheses were proposed in this study: MS and MVPA served as independent mediators in the relationship between SES and PF.

Although previous studies have examined the pairwise relationships among family SES, MS, MVPA, and PF, few have integrated these four variables into a unified analytical framework to systematically investigate the sequential mediating roles of MS and MVPA in the association between SES and PF among adults. To address this research gap and based on the above theoretical foundation, this study proposes a fourth hypothesis: MS—MVPA sequentially mediate the relationship between SES and PF. This hypothesis aims to explain the long-term mechanism through which SES influences PF in adults.

Therefore, the specific purposes of this study are as follows: (1) to examine the effects of SES on adult PF; (2) to assess the independent mediating effects of MS and MVPA between SES and PF in adults, respectively; and (3) By examining the sequential mediating effect of MS and MVPA in the relationship between SES and PF among adults to uncover the underlying pathways through which SES influences PF, thereby providing scientific evidence and policy implications for improving adults’ PF and promoting health equity.

## 2 Materials and methods

The data for this study were obtained from the 2020 Macao Special Administrative Region Physical Fitness and Health Surveillance (data of citizens aged 20–59 years), which was carried out by the Sports Bureau of the Government of the Macao Special Administrative Region from July 1 to November 30, 2020. A stratified multi-stage sampling method was adopted, with age and gender as the primary stratification variables. Other relevant factors, such as occupation, were also considered to ensure that the sample was representative and could accurately reflect the overall physical fitness and health status of Macao citizens. The survey mainly covered the physical fitness status, physical activity and exercise habits of the Macao population. The survey followed the World Declaration of Helsinki (20) and the STROBE (21) (the Reporting of Strengthening Observational Studies in Epidemiology) statement. Ethical approval for this survey was obtained from the China Institute of Sport Science (No. CISSLA-20190607).

Subjects with missing data on general demographic characteristics, physical fitness, motor behaviors, or physical activity were excluded from the study, and a total of 3,695 subjects were finally included.

### 2.1 Selection of indicators

#### 2.1.1 Socioeconomic status

SES is usually measured based on multiple dimensional indicators such as income, education, and occupation (22, 23), and some studies have used only two of these indicators to calculate SES (24). According to the data in this study, two indicators, namely education level and occupation type, were used to calculate SES, in which education level was categorized into four levels (minimum: 1 = primary school and below, maximum: 4 = postgraduate students and above); and occupation type was categorized into five levels (minimum: 1 = temporary workers, unemployed, job seekers, and unskilled labor; maximum: 5 = senior managers and senior professionals and technicians). Before conducting principal component analysis, firstly, the standardized scores of the assigned indicators were processed (*Z*-score standardization), and then the correlation of the standardized indicators was examined, and the results showed that KMO = 0.726, Bartlett’s spherical test *χ*^2^ = 314.873 (*p* < 0.01), which indicated that the data were capable of conducting principal component analysis. The principal components were then extracted according to the criterion of eigenvalues greater than 1. Only one principal component was extracted (eigenroot = 1.286), which explained 64.295% of the total variance. Under the first principal component, the factor loadings for education level and occupation type were 0.802 and 0.624, respectively. That is, SES = (0.624 × Z_education_ level + 0.802 × Z_occupation_ type)/1.286, with higher scores indicating a higher SES.

#### 2.1.2 Physical fitness

Physical fitness was measured according to the Macao Citizen’s Physical Fitness Measurement Standards Manual (2018 edition) (Adults: 20–59 years old) (25), which included a total of the following tests: (1) grip strength using an electronic grip strength meter; (2) chair sit-and-reach test using a sit-and-reach tester; (3) closed-eye one-legged stand tester to test closed-eye single-leg stand; and (4) using an electronic reaction time tester to test choice reaction time. Each test was performed twice and the best score of the 2 trials was recorded. Then, according to the scoring criteria in the manual, each indicator was converted to a 5-point scale. The total score was then calculated, with higher scores indicating better physical fitness.

#### 2.1.3 Motor skills

In this study, the number of sports participated in was used to measure MS. This was assessed through the questionnaire item “Which sports have you participated in?” A total of 34 options were provided, including basketball, football, table tennis, etc., and participants could also report other sports not listed. Each sport was counted as 1 point, with higher scores indicating a higher level of MS.

#### 2.1.4 Physical activity

Participants’ physical activity was investigated using the International Physical Activity Questionnaire Short Form (IPAQ-SF), which consists of 7 questions in which participants recall the time and frequency they were physically active in the past week in three types of physical activity: Light Physical Activity (LPA) and Moderate Physical Activity (MPA) and Vigorous Physical Activity (VPA) in the past week. The IPAQ-SF was used for the calculation of physical activity levels, and showed strong reliability in China with a Cronbach’s *α* coefficient of 0.890 (26).

### 2.2 Control variables

Referring to previous studies (27), demographic variables were included: (1) gender (1 = male, 2 = female), (2) age was transformed into a continuous variable, and (3) years of residence was transformed into a continuous variable.

### 2.3 Statistical methods

Statistical analyses were conducted using SPSS 29.0 (IBM Corp., Armonk, NY, USA). The normality of variables was tested using the Kolmogorov–Smirnov (K–S) test. Continuous variables were described as mean ± standard deviation (*M* ± SD), and categorical variables were presented as frequency and percentage [*n* (%)]. All variables were confirmed to follow a normal distribution. Independent-sample t-tests and chi-square (χ^2^) tests were used to compare differences between groups. The Harman’s single-factor test was used to assess common method bias. Pearson correlation analysis and multiple linear regression were employed to examine variable correlations and predictive relationships, respectively. Mediation effects were analyzed (28) using the PROCESS macro (Version 4.0) in SPSS, with bias-corrected bootstrapping (5,000 resamples). A 95% confidence interval (CI) was used, and mediation was considered significant if the CI did not include 0. To further examine group-level variation in the association between SES and PF, stratified regression analyses were conducted by age and gender. SES was included as the independent variable and PF as the dependent variable, with relevant confounders controlled for in all models. Statistical significance was set at *p* < 0.05.

## 3 Results

Harman’s single-factor test was used to assess the impact of common method bias. The results showed that there were three factors with eigenroots > 1, and the first factor accounted for 31.265% of the total variance, which is below the critical threshold of 40%. Therefore, common method bias was not considered a serious issue in this study (29).

### 3.1 Descriptive statistics

A total of 3,695 valid samples were obtained in this study, including 1,435 males (38.8%) and 2,260 females (61.2%). Regarding educational level, 132 participants (3.6%) had primary school education or below; 762 (20.6%) had completed secondary school; 2,251 (60.9%) had a bachelor’s degree, and 550 (14.9%) had a master’s degree or above. In terms of occupation, 665 participants (18.0%) were temporary workers, unemployed, or unskilled laborers; 152 (4.1%) were engaged in manual labor or self-employment; 1,114 (30.1%) were general management or technical personnel; 1,213 (32.8%) were middle-level management or professionals; and 551 (14.9%) were senior management or highly skilled professionals. The average years of residence was 30.4 ± 14.44 years. Significant differences in group composition were observed across age groups (*p* < 0.05) (Table 1).

**Table 1:** Basic information (*n* = 3,695).

Variables	20–29 years	30–59 years	20–59 years	Statistical magnitude	*p*
Gender, *n* (%)				10.234	<0.01
Male	760 (36.6)	675 (41.7)	1,435 (38.8)		
Female	131 (63.4)	942 (58.3)	2,260 (61.2)		
Educational level, *n* (%)				555.253	<0.01
Primary school and below	5 (0.2)	127 (7.9)	132 (3.6)		
Secondary school	207 (10.0)	555 (34.3)	762 (20.6)		
Bachelor’s degree	1,550 (74.6)	701 (43.4)	2,251 (60.9)		
Master’s degree or above	316 (15.2)	234 (14.5)	550 (14.9)		
Occupation, *n* (%)				110.801	<0.01
Temporary workers, unemployed, or unskilled laborers	335 (16.1)	330 (20.4)	665 (18.0)		
Manual workers and self-employed persons	32 (1.5)	120 (7.4)	152 (4.1)		
General management versus general professional and technical staff	626 (30.1)	488 (30.2)	1,114 (30.1)		
Middle management and middle-level professional and technical staff	768 (37.0)	445 (27.5)	1,213 (32.8)		
Senior managers and senior professional and technical staff	317 (15.3)	234 (14.5)	551 (14.9)		
Years of residence (years), Mean ± SD	24.0 ± 10.55	38.5 ± 14.68	30.4 ± 14.44	−33.445	<0.01
Total, *n* (%)	207 (56.2)	1,617 (43.8)	3,695		

Years of residence: *t*-test for independent samples, *t*; chi-square test for all other tests, *χ*^2^.

### 3.2 Correlation analysis

Correlation analysis (Table 2) showed that SES was positively correlated with MS (*r* = 0.064, *p* < 0.01), MVPA (*r* = 0.069, *p* < 0.01), and PF (*r* = 0.055, *p* < 0.01). MS were positively correlated with MVPA (*r* = 0.129, *p* < 0.01) and PF (*r* = 0.107, *p* < 0.01), while MVPA was positively correlated with PF (*r* = 0.118, *p* < 0.01), and there was a correlation between the four variables and the control variables (*p* < 0.01), except for PF and gender, where MVPA was correlated with age (*r* = 0.058, and *p* < 0.01) and years of residence (*r* = 0.049, *p* < 0.01) were positively correlated, and the rest were negatively correlated (*p* < 0.01).

**Table 2:** Correlation between variables (*n* = 3,695).

Variables	SES	Motor skills	MVPA	PF	Gender	Age	Years of residence
SES	1						
Motor Skills	0.064**	1					
MVPA	0.069**	0.129**	1				
PF	0.055**	0.107**	0.118**	1			
Gender	−0.088**	−0.103**	−0.0113**	0.005	1		
Age	−0.110**	−0.096**	0.058**	0.080**	−0.031	1	
Years of residence	−0.101**	−0.055**	0.049**	0.046**	−0.053**	0.586**	1

**p < 0.01; *p < 0.05.

### 3.3 Analysis of mediation effects

Using stepwise regression in multiple linear regression, regression analyses were performed on SES (independent variable), PF (dependent variable), MS and MVPA (mediating variables), and gender, age and years of residence (control variables).

In model 1, gender and years of residence were not correlated with PF, while age (*β* = 0.081, *p* < 0.01) was positively associated with PF; In Model 2, after SES was added to Model 1, gender and length of residence remained not associated with PF, while age (*β* = 0.086, *p* < 0.01) and SES (*β* = 0.066, *p* < 0.01) were positively associated with PF. In Model 3, after MS were added to Model 2, the control variables remained unchanged, and both SES (*β* = 0.061, *p* < 0.01) and MS (*β* = 0.115, *p* < 0.01) showed significant positive associations with PF. In Model 4, after MVPA was further added to Model 3, age and length of residence remained not associated with PF, while gender (*β* = 0.035, *p* < 0.01), SES (*β* = 0.055, *p* < 0.01), MS (*β* = 0.103, *p* < 0.01), and MVPA (*β* = 0.099, *p* < 0.01) were all positively associated with PF (see Table 3).

**Table 3:** Regression results of the chain mediation model (*n* = 3,695).

Variables	Model 1			Model 2			Model 3			Model 4		
	b	SE	*β*	b	SE	*β*	b	SE	*β*	b	SE	*β*
Gender	0.054	0.118	0.008	0.098	0.118	0.014	0.181	0.118	0.026	0.245	0.118	0.035**
Age	0.025	0.006	0.081**	0.027	0.006	0.086**	0.03	0.006	0.097*	0.028	0.006	0.091
years of residence	0.001	0.005	−0.001	0.001	0.005	0.003	0.001	0.005	0.003	0.001	0.005	0.001
SES				0.415	0.105	0.066**	0.383	0.104	0.061**	0.345	0.104	0.055**
Motor Skills							0.176	0.025	0.115**	0.157	0.026	0.103**
MVPA										0.001	0.001	0.099**
F	7.801			9.787			17.419			20.523		
R^2^	0.006			0.011			0.024			0.033		

b, unstandardized coefficient; SE, Standard Error; *β*, Standardized Regression Coefficient; **p < 0.01; *p < 0.05.

**Figure 1:**
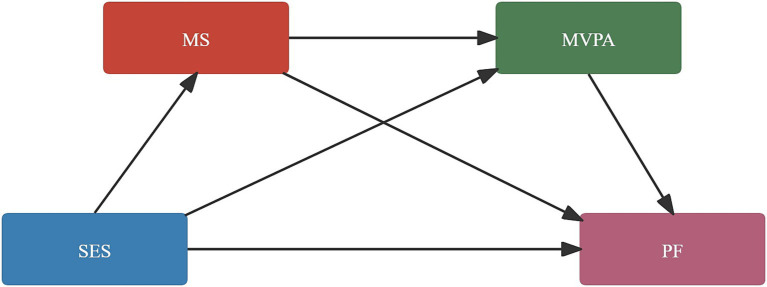
Relationship between SES, PF, motor skills and MVPA. SES, socioeconomic status; MS, motor skills; MVPA, moderate to vigorous physical activity; PF, physical fitness.

The sensitivity analysis showed that the unstandardized regression coefficient for the specified path was 0.407, with a standard error of 0.105 and a bootstrap bias of 0.003, representing 0.79% of the estimate. These findings suggest that the estimate is stable and reliable. The 95% bootstrap confidence interval (0.216 to 0.629) did not include 0, indicating that the path coefficient was statistically significant. The relatively narrow confidence interval and small standard error suggest good precision and robustness of the estimate (see Figure 1).

Within the age subgroups, SES was significantly positively associated with PF among individuals aged 30–59 years (*β* = 0.078, *p* < 0.05) and also showed a positive association among those aged 20–29 years (*β* = 0.053, *p* < 0.05). However, the effect size was relatively smaller in the younger group, suggesting that the influence of SES on PF may be more pronounced in middle-aged adults. In the gender subgroups, SES was significantly positively associated with PF in both males (*β* = 0.062, *p* < 0.05) and females (*β* = 0.067, *p* < 0.05), with slightly higher standardised coefficients observed in females. In summary, the positive association between SES and PF was consistent across both age and gender groups, but relatively stronger among individuals aged 30–59 years and among females (see Table 4).

**Table 4:** Stratified analysis (*n* = 3,695).

	b	SE	*β*
20–29 years	0.343	0.145	0.053*
30–59 years	0.468	0.151	0.078*
Male	0.417	0.181	0.062*
Female	0.403	0.128	0.067*

b, unstandardized coefficient; SE, Standard Error; *β*, Standardized Regression Coefficient; **p < 0.01; *p < 0.05.

**Figure 2:**
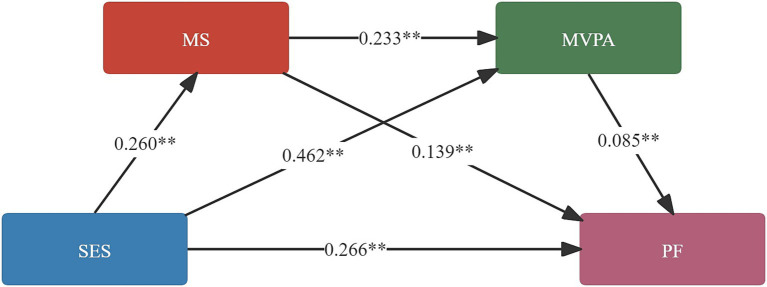
Chain mediation effect. SES, socioeconomic status; MS, motor skills; MVPA, moderate to vigorous physical activity; PF, physical fitness; the coefficients in the figure are standardised coefficients.

Mediation analysis was conducted using the SPSS macro Process 4.0. The mediation results are presented in Table 4. An indirect effect of SES on PF was found, with the 95% confidence interval not containing zero, indicating that both mediating variables exerted significant mediation effects in the relationship between SES and PF. The total mediation effect consisted of three indirect pathways: SES → MS → PF, with an effect size of 0.0361 [95% CI = (0.0149, 0.0629)]; SES → MVPA → PF, with an effect size of 0.0392 [95% CI = (0.0172, 0.0682)]; SES → MS → MVPA → PF, with an effect size of 0.0051 [95% CI = (0.0020, 0.0097)]. The proportion of the total effect accounted for by these three pathways was 10.42, 11.32, and 1.47%, respectively (see Table 5 and Figure 2).

**Table 5:** Standardized direct and indirect pathways (*n* = 3,695).

Mechanism	Effect	BootSE	95%CI		Contribution rate (%)
			LLCI	ULCI	
Total effect	0.3463	0.1041	0.1422	0.5505	100.00
Direct effect	0.2659	0.1034	0.0631	0.4687	76.78
Total indirect effect	0.0804	0.0189	0.0469	0.1214	23.22
SES-Motor Skills-PF	0.0361	0.0122	0.0149	0.0629	10.42
SES-MVPA-PF	0.0392	0.0130	0.0172	0.0682	11.32
SES-Motor Skills-MVPA-PF	0.0051	0.0019	0.0020	0.0097	1.47

5000 Bootstrap samples.

## 4 Discussion

Due to Macao’s unique geographical location, historical background, and economic conditions, it differs significantly from Mainland China. Therefore, this study explored the impact of SES on the PF of adults in Macao, constructed a chain mediation model involving SES, PF, MS, and MVPA, and tested its validity based on the research objectives.

### 4.1 Direct effect of SES on PF

This study found that SES positively predicts PF among adults in Macao, accounting for 23.22% of the effect size, which is highly consistent with previous studies (30–32). This result further validates the critical role SES plays in individual PF levels. Mechanistically, individuals with higher SES generally possess more abundant economic resources, enabling access to superior nutrition and more convenient, advanced healthcare services (33, 34). Compared to mainland China, Macau has limited land resources and a high degree of spatial concentration of quality community facilities (35). As a result, families with higher SES are more likely to reside in areas with better access to supportive infrastructure (e.g., fitness trails, sports venues, and green parks) and strong neighborhood networks (36). These factors provide favorable conditions that facilitate the enhancement of PF. Additionally, Macao implements 15 years of compulsory education, resulting in a relatively high overall educational attainment among its residents. Higher educational attainment is often associated with greater health literacy and improved ability to access health information, prompting individuals to adopt healthier behaviors and reduce risk-taking (37, 38). Individuals with higher SES are more likely to actively engage in health risk management and lifestyle interventions, thereby developing positive physical activity patterns and healthy habits. Moreover, they have greater access to global health concepts and modern lifestyle trends—such as evidence-based fitness practices and functional nutrition—which further contribute to improvements in their PF levels (39).

The subgroup analysis of this study showed that the effect of SES on PF was more pronounced among middle-aged women. This may be attributed to the fact that, within adult populations, individuals from higher SES backgrounds tend to be more motivated to maintain their health as they age (40). Furthermore, they place greater emphasis on the pleasurable experiences associated with physical activity, making the impact of SES on PF more evident in middle-aged groups (41). Although existing literature suggests that men generally exhibit superior physical function compared to women (42) and that adult women often bear a greater family burden (43), women from high-SES households tend to prioritize physical health and derive greater psychological and physical satisfaction from exercise. As a result, they may demonstrate a stronger willingness to enhance their PF.

### 4.2 Mediating effects of MS

This study found that SES positively predicts MS among adults in Macao, which aligns well with existing literature (44). Higher SES provides individuals with more abundant opportunities for physical activity, such as access to professional training and the ability to purchase sports facilities—both of which are critical for fostering interest in and development of MS (45). Additionally, individuals with higher SES often enjoy more flexible work schedules and experience less financial stress. These findings should be interpreted in the context of Macao’s unique sociocultural environment, allowing them to allocate more leisure time for physical exercise. This, in turn, supports consistent engagement in skill-based sports activities such as tennis, swimming, or dance (46). Furthermore, MS were found to be a positive predictor of PF in adults, consistent with previous studies (47). As the foundation for performing daily activities and structured physical exercise, higher levels of MS promote more active and sustained participation in physical activity. This ultimately contributes to the improvement of key components of PF, including cardiovascular health, muscular strength, and flexibility (48).

### 4.3 Mediating effects of MVPA

This study found that SES positively predicts MVPA among adults in Macao, which is consistent with previous research (49). Compared to individuals with higher SES, those with lower educational attainment may have reduced awareness of the benefits of physical activity (50), which can weaken their exercise self-efficacy. In addition, limited financial resources may restrict access to fitness equipment or sports facilities among low-SES groups (51), thereby hindering their engagement in MVPA. Moreover, our findings confirmed a significant positive association between MVPA and PF in adults, which is in line with other studies (14, 52). Adequate physical activity plays a key role in maintaining and enhancing essential components of PF, including cardiorespiratory endurance (53), muscular strength (54), and body composition (55). Particularly in Macao, where population ageing is becoming increasingly prominent (56), MVPA as a modifiable and promotable lifestyle behavior plays a critical role in slowing physical function decline and enhancing the overall health quality of residents.

### 4.4 Chain mediation effect of MS-MVPA

The chain mediation model proposed in this study: SES → MS → MVPA → PF—illustrates the sequential mediating roles of MS and MVPA in the relationship between SES and PF among adults. Higher SES provides individuals with more resources, supportive environments, and discretionary time, all of which lay a solid foundation for the development and acquisition of MS, aligning with their pursuit of better physical health. In Macao, where the urban structure is compact, the pace of life is fast, and work-related stress is generally high, individuals with higher SES are more likely to have flexible schedules and greater disposable income. These advantages enable them to participate more easily in sports that require a certain level of technical skill. The effective acquisition of MS significantly enhances individuals’ exercise motivation and self-efficacy (57), which in turn drives greater engagement in MVPA. Additionally, Macao’s hilly terrain, characterized by narrow streets and numerous slopes in urban areas, inherently encourages active modes of transportation, thereby contributing to increased levels of MVPA. Sufficient MVPA not only directly contributes to the improvement of PF but also reinforces health identity. Through consistent and scientifically guided physical activity over time, individuals are more likely to internalize lifelong exercise habits, forming an organic chain of need–motivation–behavior–health (58). The preventive potential and modifiability of MVPA highlight its significant value for public health promotion. In summary, MS function as a pathway through which the advantages conferred by SES promote MVPA across multiple dimensions, ultimately laying the foundation for improvements in adult PF.

### 4.5 Limitations

The present study used a simplified approach to assess SES, incorporating only two indicators: education and occupation. This limited measurement may have underestimated the influence of broader economic factors. In addition, MVPA was assessed using self-reported questionnaire data, which is subject to recall bias. Future research is encouraged to incorporate accelerometer-based measures to improve the accuracy of physical activity assessment. Furthermore, MS were evaluated based on the number of sports disciplines in which participants engaged. While this reflects the breadth of skill exposure, it does not capture the depth or quality of motor performance. Although this method offers practical advantages, it may underestimate individuals’ true motor competence.

## 5 Conclusion

This study developed and validated a chained mediation model in which SES influences adults’ PF through MS and MVPA. The results indicate that higher SES, through its advantages, facilitates the development of MS in adults, which in turn promotes greater engagement in MVPA, ultimately contributing to improved PF levels. These findings emphasize the pivotal role of SES in shaping PF and highlight MS and MVPA as critical mediating pathways. The study provides a novel perspective for understanding the complex relationship between SES and PF and offers valuable theoretical guidance for designing targeted health interventions for adults in Macao.

## Data Availability

The data analyzed in this study is subject to the following licenses/restrictions: data presented in this study are available on request from the corresponding author. The data are not publicly available due to privacy. Requests to access these datasets should be directed to YZ, zhangyanfeng0310@126.com.
